# Active Aging for Undergraduate University Students: Lessons From Student See Me at 70 Course Reflections

**DOI:** 10.1093/geroni/igaa057.019

**Published:** 2020-12-16

**Authors:** Emily Roberts

**Affiliations:** Oklahoma State University, Stillwater, Oklahoma, United States

## Abstract

Demographic factors tell us that the number of adults age 65 and older will nearly double by 2030. A holistic and proactive approach to providing positive outcomes in aging requires integrated strategies focusing on providing organizational structures to support this demographic shift. Active aging is a framework first developed by the World Health Organization optimizing opportunities for health, participation and security in order to enhance quality of life as people age. Active aging allows people to realize their potential for physical, social and mental wellbeing throughout the life course. This presentation shares outcomes from a university honors active aging seminar entitled Active Aging for L.I.F.E. in which students were provided with information on a wide range of topics in order to build their personal understanding of the relationships between Longevity, Independence, Fitness and Engagement. Student were then asked to develop a final paper entitled See Me at 70, in which they were to use the course content to develop a narrative of power and inspiration about their future self at age 70. What tactics would be used through their lifespan to develop a behavior and belief system which would enable them to live their life to the fullest both physically and cognitively? How would they respond to the challenges of aging and turn them in to opportunities? This presentation shares key themes of the student work, including the relationships between independence and interdependence, the connections between physical and cognitive health, maintaining purpose and facing unforeseen challenges with a positive attitude.

